# Microneedle Arrays Combined with Nanomedicine Approaches for Transdermal Delivery of Therapeutics

**DOI:** 10.3390/jcm10020181

**Published:** 2021-01-06

**Authors:** Vahid Alimardani, Samira Sadat Abolmaali, Gholamhossein Yousefi, Zahra Rahiminezhad, Mehdi Abedi, Alimohammad Tamaddon, Samad Ahadian

**Affiliations:** 1 Department of Pharmaceutical Nanotechnology, School of Pharmacy, Shiraz University of Medical Sciences, Shiraz 71348-45794, Iran; v_alimardani@sums.ac.ir (V.A.); rahiminezhad.zahra@yahoo.com (Z.R.); mehdi.abedi.a@gmail.com (M.A.); amtamadon@gmail.com (A.T.); 2 Center for Nanotechnology in Drug Delivery, Shiraz University of Medical Sciences, Shiraz 71348-45794, Iran; ghyousefi@sums.ac.ir; 3 Terasaki Institute for Biomedical Innovation, Los Angeles, CA 90024, USA

**Keywords:** microneedle arrays, drug delivery, immunotherapy, vaccination, gene delivery, nanomedicine

## Abstract

Organic and inorganic nanoparticles (NPs) have shown promising outcomes in transdermal drug delivery. NPs can not only enhance the skin penetration of small/biomacromolecule therapeutic agents but can also impart control over drug release or target impaired tissue. Thanks to their unique optical, photothermal, and superparamagnetic features, NPs have been also utilized for the treatment of skin disorders, imaging, and biosensing applications. Despite the widespread transdermal applications of NPs, their delivery across the stratum corneum, which is the main skin barrier, has remained challenging. Microneedle array (MN) technology has recently revealed promising outcomes in the delivery of various formulations, especially NPs to deliver both hydrophilic and hydrophobic therapeutic agents. The present work reviews the advancements in the application of MNs and NPs for an effective transdermal delivery of a wide range of therapeutics in cancer chemotherapy and immunotherapy, photothermal and photodynamic therapy, peptide/protein vaccination, and the gene therapy of various diseases. In addition, this paper provides an overall insight on MNs’ challenges and summarizes the recent achievements in clinical trials with future outlooks on the transdermal delivery of a wide range of nanomedicines.

## 1. Introduction

Skin is responsible for enclosing and protecting the human body against the invasion of microorganisms, allergens, toxins, and UV irradiation [1]. Meanwhile, it serves as a pathway for administering therapeutics [2]. Compared to oral, nasal, intramuscular, and intravenous delivery routes, transdermal delivery (TDD) has immense advantages, such as pain-free administration over hypodermic injection, patients’ compliance, and self-administration [3]. Transdermal drug delivery systems (TDDSs) not only contribute to the continuous transport of therapeutic agents through the skin, but they also help the agent to overcome certain barriers (such as first-pass metabolism) and promote the transport of agents with low solubility and bioavailability [4]. However, passive skin transport is restricted to small lipophilic molecules.

**Figure 1:**
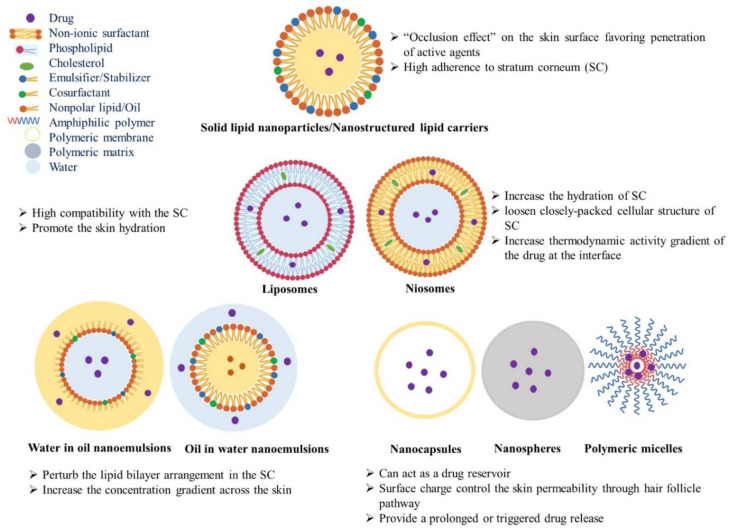
Vesicular, lipid and polymer-based nanoparticles (NPs) currently used in nanomedicine and their underlying mechanisms for enhanced transdermal delivery (TDD).

Nanotechnology has been explored in TDD to enhance the permeation of therapeutic agents through the skin. Due to their small size and large surface area, nanoparticles (NPs) can deliver drugs across the stratum corneum (SC) without disrupting the skin barrier function via transcellular or trans-appendageal pathways [5]. Various types of nanocarriers have been reported in transdermal drug delivery. Carrier-free nanomedicines have shown low toxicity, enhanced drug solubility, and dissolution [6]. Lipid vesicles have gained interests due to their ability to deliver drugs by “free drug mechanism”. The released drug molecules can permeate into the skin due to an effective penetration mechanism of carrier component(s) interacting with SC lipids. Thanks to their miscibility with SC lipids, liposomes have shown great promise in TDD. However, their adherence to corneocytes can result in the leakage of encapsulated therapeutic agents at the superficial layers of skin. Hence, several vesicular carriers, such as Niosomes, Ethosomes^®^, Transfersomes^®^, Invasomes^®^, Vesosomes^®^, Proliposomes, and Pharmacosomes^®^ have been introduced as reviewed before [5]. Nanoemulsions, either water-in-oil (W/O) or oil-in-water (O/W) can also enhance TDD due to their small droplet size and the constituent surfactants, which destabilize the SC lipid bilayer (Figure 1) [5]. Lipid NPs, especially nanostructured lipid carriers (NLCs), can efficiently load insoluble drugs and thanks to their high adherence to the SC, they can facilitate the drug penetration into deeper layers of the skin [7,8]. Polymeric systems, such as a polymer–drug conjugate, nanospheres, dendrimers, polymeric micelles, polymeric vesicles and microcapsules can control the release and skin retention of therapeutic materials for a prolonged period (Figure 1) [9,10,11]. Inorganic NPs and carbon nanomaterials, such as superparamagnetic iron oxides (SPIONs), titanium dioxide (TiO_2_), zinc oxide (ZnO), gold (AuNPs), silver (AgNPs), quantum dots (QDs), mesoporous silica nanoparticles (MSNs), carbon nanotubes (CNTs), graphene oxide (GO), carbon nanofibers (CNFs), and fullerenes have been recently applied in not only TDD, but also in the diagnosis or treatment of skin disease [12,13]. Despite recent progress in nanomaterials, the safety and biological fate of these NPs should be explored to develop efficient TDDSs. To improve the skin penetration of NPs, various techniques, including physical methods as well as chemical permeation enhancers, have been explored. Iontophoresis [14], dermaportation [15], sonophoresis [16], and microneedle arrays (MNs) [17] have been applied in the delivery of not only small and large therapeutic agents, but also various NPs through the stratum corneum (SC), the outermost and nonviable barrier layer of the skin [12].

With an emergence of the microfabrication techniques in mid-1990s, MNs have received enormous attention in TDD applications [18]. Since the advent of MNs, they have shown promising results due to their minimally invasive penetration into the SC, which promotes the delivery of therapeutic agents. In recent years, different types of MNs, such as solid, hollow, coated, dissolving, and hydrogel-forming MNs, have been developed for transdermal drug delivery and cosmetic applications [19,20]. This review highlights the significance and efforts made in the design of different MNs. In the following sections, the current advancement in the MN-assisted TDD of NPs will be discussed with a particular focus on cancer chemo- and immuno-therapy, photodynamic therapy (PDT), photothermal therapy (PTT), vaccination, therapeutic protein delivery, and gene therapy. Finally, current clinical trials as well as challenges and future outlook in this research area will be addressed.

## 2. MNs: An Advancement in Hypodermic Needles

Even though hypodermic needles have been the gold standard for drug delivery and vaccination, they suffer from several drawbacks such as pain, needle phobia, hazardous sharp waste, and injury [21,22]. Hence, MNs have been developed to overcome the limitations of hypodermic needles. MNs are minimally invasive micron-sized needles, clustered on a solid base or patch. MNs have recently gained significant attention as a popular way of delivering macromolecules as well as small molecules across the skin due to their low cost, painlessness, and self-administration [23]. MNs can be classified according to their structures, delivery approaches, fabrication techniques, shapes, and constituent materials. Hence, they can be classified in more than one class. For example, MNs can be hollow based on their structure or delivery approach and at the same time they can be fabricated from metals, ceramics or biodegradable polymers [24]. The MNs can be classified based on delivery strategies as follows [19]:

(A) Poke-and-patch approach to apply tens to tens of thousands of MNs as a pore-forming pretreatment. Afterward, a conventional drug formulation is applied on the skin surface.
(B) Coat-and-poke approach, which consists of a water-soluble drug coating on solid MNs. Drug coating is dissolved and simply deposited within the skin during its administration.
(C) Poke-and-release approach in which water-insoluble MNs are injected into the skin. The encapsulated therapeutic agent is slowly released, while the patch remains on the skin after its application.
(D) Poke-and-flow approach, which is characterized by a hole in the structure (center or side) of each microneedle to enable the drug flow across the skin.
(E) Poke-and-dissolve approach, which uses biodegradable or water-soluble drug-encapsulated MNs. The MNs dissolve and release their loaded therapeutic agents into the skin. Table 1 summarizes different delivery strategies, advantages, disadvantages, and applications of MNs.

**Figure 2:**
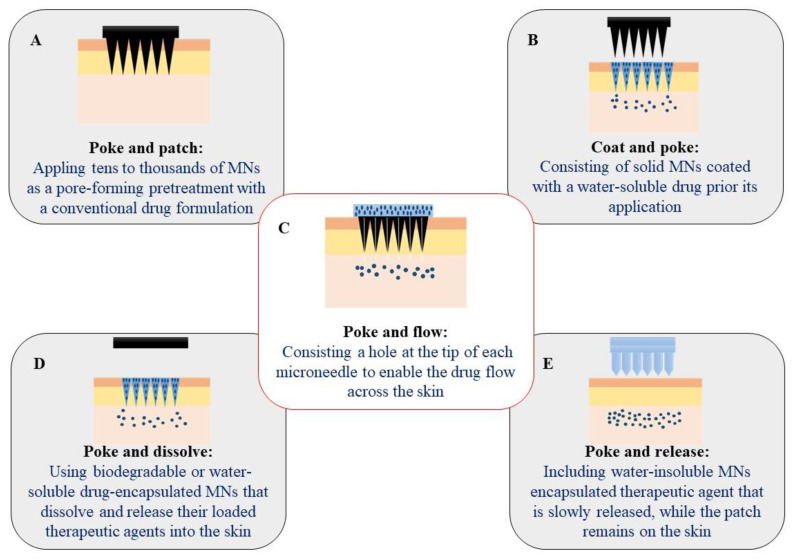
A schematic representation of delivery approaches using various types of microneedle arrays (MNs): (**A**) poke-and-patch (solid MNs), (**B**) coat-and-poke (coated MNs), (**C**) poke-and-flow (hollow MNs), (**D**) poke-and-dissolve (dissolvable MNs), and (**E**) poke-and-release (hydrogel forming MNs).

As shown in Figure 2, MNs can be classified based on delivery strategies as follows:

Solid MNs—solid MNs use the “poke-and-patch” approach to deliver drugs or macromolecules [25]. The first MNs-based TDDS was fabricated based on silicon MNs to simplify in vitro calcein delivery across the excised human skin [26]. Silicon is a widely used material for micromachining MNs, as it can be easily processed to make MNs with different sizes and morphologies. Despite the great advantages of silicon in the fabrication of solid MNs, several limitations, such as cost, a long and complex manufacturing process, fragility, and the biocompatibility concerns have led to the development of novel materials for the fabrication of solid MNs. To date, various biocompatible, strong, and easily synthesized materials have been applied to facilitate the penetration of proteins, hormones, and vaccines into the skin, as presented in Table 1 [24]. For more detailed information, refer to excellent reviews on MN-assisted vaccination and the applications of solid MNs [27,28].

Hollow MNs—drug formulations that can be delivered by the “poke-and-flow” approach use hollow MNs with different height and geometries [29]. Silicon and metals are the main constituent materials to make hollow MNs (Table 1). Hollow MNs can deliver relatively high quantities of therapeutic agents by diffusion, pressure- or electrically- driven flow. Moreover, in combination with the iontophoresis technique, they can actively displace charged drugs or particles in a well-controlled manner [30]. To date, various therapeutic agents, such as proteins and vaccines as well as diagnostic agents have been delivered into the skin via hollow MNs (Table 1). Interested readers are directed to excellent reviews on this topic [27,28].

Coated MNs—coated MNs are fabricated by coating a drug layer on solid MNs [25]. For efficient drug delivery, the formulations should be uniformly coated, which highly depends on the wetting angle and spread of the drug formulation. Moreover, the drug formulation ought to be stable, water-soluble, and strong enough to maintain the coated material during the insertion into the skin. Layer-by-layer coated MNs have also been fabricated by the alternate dipping of metallic, polymeric, or silica MNs into a solution containing oppositely charged agents [31,32]. For example, Schipper et al. developed layer-by-layer pH-sensitive coated silicon MNs for the delivery of diphtheria toxoid (DT) as a promising vaccination strategy. First, the surface of MNs was chemically modified with pyridine functional groups to achieve a positive surface charge at a pH of 5.8. Then, multiple alternating layers of DT with a negative charge and positively charged N-trimethyl chitosan (TMC) were applied by a layer-by-layer coating procedure. It was shown that the increasing the number of DT/TMC bilayers resulted in a higher immune response with lower DT content [33]. As presented in Table 1, various formulations have been coated on MNs to facilitate the skin permeation of therapeutic agents, including vaccines, proteins, and hormones. More detailed information about coated MNs can be found in other publications [34,35].

Dissolvable/biodegradable MNs–nowadays, polymeric materials have been widely used in the fabrication of MNs, owning to their biocompatibility and low cost. Apart from their biocompatibility, some of them are biodegradable. Dissolvable MNs (dMNs) are completely dissolved in the skin, quickly leaving no biohazardous sharp wastes after use. They act based on a poke-and-dissolve approach in which the drugs are encapsulated in a safe, inert, and water-soluble polymer or sugar-based matrix [25]. Biodegradable MNs are formed using various types of biodegradable polymers, such as chitosan, hyaluronic acid (HA), polylactic acid (PLA), polyglycolic acid (PGA) or poly (lactide-co-glycolide) (PLGA) to form a matrix, that are degraded after the skin insertion [24]. The biodegradable MNs release the loaded cargos for months by diffusion and polymer erosion mechanisms [36]. To achieve high drug loading into dMNs and enhance their mechanical strength, several novel dMNs, such as double-layer, pedestal, and separable arrowhead dMNs have been fabricated [37]. Separable arrowhead MNs have also been developed on a metal support and a dissolvable tip to overcome the drawbacks associated with the low mechanical strength of dMNs and the biohazardous sharp waste of solid MNs [38,39]. The sharp-tipped polymer arrowhead MNs were able to insert into the skin and release their drug content after being dissolved in situ [40]. Among several advantages, dMNs do not require any pump or patch. In addition, they do not leave any sharp waste and their fabrication cost is fairly low. However, there are some issues related to dMNs, such as low mechanical strength and penetration ability, the delivery of small doses, and the need for drug reformulation. Interested readers are encouraged to refer to excellent reviews on MN-assisted drug delivery [41,42]. In addition to modifying the drug loading property of MNs, a drug release profile from MNs can be modified via the incorporation of bioresponsive NPs, resulting in the emergence of a novel class of MNs named “bioresponsive MNs”. The bioresponsive MNs can release the therapeutic agents in response to physiological signals, which in turn can modulate the therapeutic effectiveness and drug toxicity profiles. To date, some dissolvable or biodegradable polymers, such as polyvinylpyrrolidone (PVP) and modified HA have been reported in combination with NPs for the development of MNs responsive to pH, glucose, and enzymes [43,44,45].

Hydrogel-forming MNs—hydrogel-forming MNs are fabricated from polymers, which can rapidly swell upon insertion into the skin [46,47]. The MNs absorb the body fluid into their three-dimensional matrix, resulting in a controlled release of the loaded drug into the skin via their created microconduits without any measurable polymer residuals after removal [48,49]. To date, hydrogel-forming MNs, including PEG-crosslinked poly(methyl vinyl ether-co-maleic acid) [50], silk fibroin and phenylboronic acid/acrylamide [51], poly (methyl vinyl ether-co-maleic acid)/pectin [52], and poloxamer [53] have been intended for drug delivery and diagnostic applications. They are usually fabricated by the micro-molding process involving a light source [54].

Taken together, different types of MNs have shown promising results in the delivery of therapeutic agents; however, there are still some limitations for the efficient biomedical applications as presented in Table 1. Although MNs locally deliver therapeutic agents, there is still a need for precise control over drug release and the fate of MNs in vivo.

## 3. Potential Applications of MN Combination with NPs

Nowadays, numerous NP formulations have been synthesized and explored for drug delivery to carry therapeutic agents, such as proteins, vaccines, and nucleic acids [90,91,92]. NPs exhibit numerous benefits, including unique size-dependent physicochemical properties [24,93], the protection of payload against chemical or proteolytic degradation, controlled release over prolonged times [94] as well as targeted delivery to specific parts of the body; hence, reducing the side effects [95]. To date, various administration routes (e.g., oral, nasal, subcutaneous, and IV injections) have been applied for the delivery of nanomedicines. The IV injection benefits from the fast onset of operation and high plasma level. Nevertheless, injections are associated with pain, infections (due to the poor maintenance of sterile conditions), and require a skilled person for administration [96,97], thereby alternative administration routes are needed for the more efficient and controlled delivery of active agents. TDD is a promising administration route that can minimize the complications associated with oral or IV drug administration. NPs are designed to affect the SC barrier function by modulating their special physicochemical properties. Due to their small size and large surface area, NPs can deliver drugs across the SC without disrupting the skin barrier function via transcellular or trans-appendageal pathways [8]. Carrier-free nanomedicines have shown low toxicity, high drug loading, enhanced solubility, and a dissolution rate [9]. However, due to presence of the SC, the translocation of NPs across the skin is still an enormous challenge. Consequently, the emergence of new strategies or the combination of present techniques can lead to overcoming the mentioned limitations.

**Figure 3:**
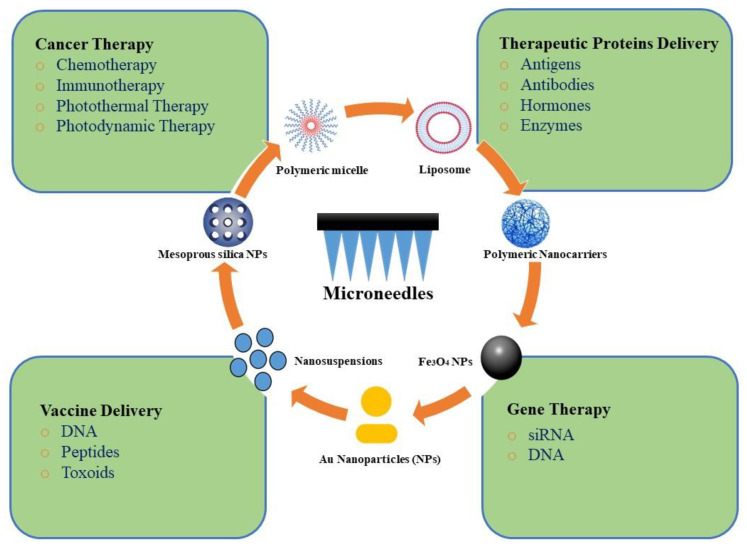
Combinatorial applications of microneedles (MNs) with nanoparticles (NPs).

Currently, MN technology has provided a versatile platform to increase the TDD of NPs in a minimally invasive way (Figure 3) [98]. In recent years, the intradermal delivery of NPs using MNs has gained a significant interest due to their unique ability: (i) to facilitate transdermal administration, (ii) to deliver both hydrophilic and lipophilic therapeutic agents, (iii) to provide the homogeneous distribution of NP-based drug reservoirs into the skin, (iv) to combine therapeutic and diagnostic agents into one unique structure giving rise to a theranostic system. Table 2 summarizes a list of reports on MN’s applications in the TDD of NPs. A quick look at the research findings suggests a synergistic enhancement in skin permeation upon combining MNs with NPs due to the reversible barrier disruption function of MNs and subsequent controlled delivery of NPs [68]. For example, Ramadan et al. developed lamivudine-loaded polylactic-co-glycolic acid (PLGA) NPs and investigated their TDD in combination with stainless steel MNs. They showed that such a prolonged release system could overcome the short half-life of the drug. Furthermore, skin treatment with MNs can result in a two-fold enhancement in the steady-state flux, offering new transport pathways to increase the TDD of the drug-loaded NPs [99]. In another study, a layer of PLGA NPs containing vitamin D3 was coated on stainless steel MNs [100]. The MNs pierced the skin layer acting as a physical enhancer for drug molecules to be localized within the dermal region. Therefore, the dual delivery approach combining nanotechnology and MNs can improve TDD while promoting the sustained release of therapeutic agents. However, this action depends on the particle size, MN-induced pore size, and the skin ability to recover. Compared with drug-loaded NPs, a small-molecule drug is expected to penetrate through the pores left by MNs in the skin [101]. Moreover, compared with micron-sized particles, drug-loaded NPs can more easily pass the skin barrier through the MN pores. More recently, nanomedicine has received great attention for the treatment of various diseases using different approaches, such as chemotherapy, protein delivery, and gene therapy due to enhanced tumor accumulation, controlled cargo delivery, and increased intracellular uptake by NPs [102]. In the following sections, the MN applications in the TDD of NPs will be discussed in various fields including chemotherapy, immunotherapy, PTT, PDT, protein, vaccine, and gene delivery.

### 3.1. MN-Assisted NP Delivery in Cancer Chemotherapy

Despite the efforts made to develop new cancer treatment strategies, chemotherapy has remained an important therapeutic modality. Numerous nanomaterials have been investigated as drug carriers to enhance the therapeutic efficacy and bioavailability of anti-cancer agents. Nonetheless, the systemic administration of anti-cancer therapeutics can trigger serious side effects [120]. Using the local TDD of anti-cancer agent-loaded NPs, we can prevent systemic toxic effects. However, there are many challenges in TDD due to the SC layers preventing the penetration of NPs. This challenge can be addressed by MNs, which can offer a minimally invasive TDD method. For example, PLGA NPs encapsulating doxorubicin (DOX) was coated on stainless steel MNs for localized drug delivery to the oral cavity tumors [121]. As MNs does not involve fluid injection, this method minimizes drug clearance from the tumor site into the systemic circulation due to the convection process. After inserting MNs, PLGA NPs were deposited at the MNs’ insertion site. Unlike hypodermic needles, which resulted in a significant loss of the injected volume, MNs produced uniform drug distribution in a porcine cadaver buccal tissue. In another study, MNs mediated the TDD of cisplatin-loaded lipid NPs for cancer treatment [122]. In this work, tumor-targeting pH-responsive lipid NPs were applied as a carrier for cisplatin encapsulation. The encapsulation led to a substantial increase in the cisplatin solubility and improved in vitro antitumor efficiency. The antitumor efficiency of MNs embedding cisplatin NPs was compared with the cisplatin loaded MNs in a xenograft mice model of head and neck tumor. The result showed an enhancement in the treatment outcomes of the NP-embedded MNs, though no comparison was made with cisplatin-loaded NPs. Furthermore, no serum platinum, hepatotoxicity, pulmonary toxicity, and nephrotoxic effect were detected, indicating the biosafety of the MN-assisted delivery of cisplatin NPs. Therefore, the combination of MNs and NPs containing chemotherapy agents can offer promising cancer treatment opportunities by improving antitumor effects and minimizing the systemic toxicity.

### 3.2. MN-Assisted NP Delivery in Cancer Immunotherapy

As an immune reactive component, skin tissue could be a site for an efficient immune response against a wide range of antigens. Different immune cells including the dendritic cells (DCs), Langerhans cells (LCs), and antigen-presenting cells (APCs) are involved in an efficient immune response. A high density of these cells, as well as their accessibility, has highlighted skin as one of the most important vaccination sites [123]. In recent years, researchers have explored therapeutic vaccines and immunotherapies in the scope of cancer via intradermal administration. For instance, some studies in animal models and patients suggest that intradermal vaccination can improve the antitumor responses in melanoma and prostate cancers [124,125].

**Figure 4:**
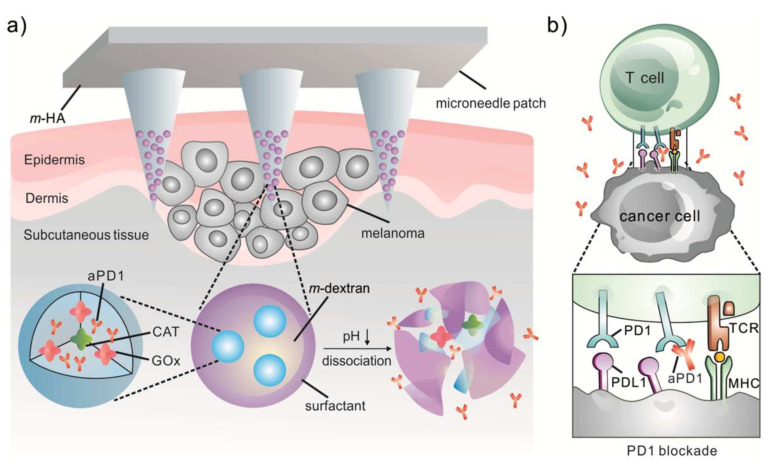
Schematic preparation of the aPD1 containing hyaluronic acid microneedles (HA MNs) for the prevention of skin cancer: (**a**) MNs containing the pH-sensitive dextran nanoparticles encapsulating catalase/glucose oxidase (CAT/GOx) enzyme system that convert the blood glucose to gluconic acid and leading to aPD1 sustained release; (**b**) aPD1 released from the MNs blocks the PD-1 receptor, which resulted in activating the immune cells to kill skin cancer cells; CAT and GOx stand for the catalase and glucose oxidase, respectively. Reprinted with permission from [45].

Considering the potential of MNs to improve percutaneous delivery, MN-mediated transcutaneous immunization can be an effective method for the stimulation of immune responses. For instance, dMNs consisting of poly (methyl vinyl ether) and maleic anhydride (PMVE/MA) laden with PLGA NPs-containing model antigen (ovalbumin, OVA) was utilized to target LCs. This approach has shown proper in vivo protection against melanoma (expressing B16 antigens) and para-influenza in a murine model by activating antigen-specific CD8+ T lymphocytes in tumors and viruses, respectively. Moreover, this study showed that antigen encapsulation in NPs can prolong the antigen’s retention time in the skin and enhance the antigen stability in MNs. Therefore, this approach could provide proper antigen protection in the case of OVA-expressed melanoma cells [126]. A few studies have also reported the application of MNs in combination with NPs for cancer vaccination. They have shown antitumor immunity in preclinical models applying MNs for the topical administration of cancer cell antigens, encoded in plasmids or obtained from tumor cell culture, which were loaded in polymeric NPs [127,128]. Indeed, a combination of MNs with NPs to deliver checkpoint inhibitors or immunosuppressive enzymes can prolong their retention time in the tumor and potentially reduce their side effects compared with the conventional administration [44]. To provide an enzyme-responsive drug release for cancer immunotherapy, Wang et al. integrated HA MNs with pH-sensitive dextran NPs containing anti-programmed death-1 (aPD1) and GOx. The blood glucose is catalyzed to gluconic acid by GOx, producing an acidic environment that causes the disintegration of NPs and subsequently, aPD1 release (Figure 4). It was shown that the single administration of the MN patch prevented the tumor’s proliferation in a mouse melanoma model, which was determined to be superior over the intratumoral injection of free aPD1 (at the same dose) or the aPD1-laden MNs without GOx-triggered degradation [45].

### 3.3. MN-Assisted NP Delivery in Photothermal Therapy

Photothermal therapy (PTT) with near-infrared (NIR) irradiation is an interesting alternative technique for chemotherapy that can be combined with conventional chemotherapy to promote a synergistic effect. In PTT, the photothermal agents convert light to heat, which can induce thermal cell injury, membrane damage, and protein denaturation [129]. Furthermore, PTT not only inhibits solid tumor growth but also triggers the immune response. Compared to existing cancer therapies, PTT can be remotely controlled, leading to low systemic toxicity and adverse effects [130]. However, with an increase in the tissue’s depth, a decreasing dose of light can reach the photothermal agent, so PTT alone fails in a complete tumor elimination. Therefore, many delivery systems combining chemotherapy and PTT have been developed to obtain synergetic effects.

**Figure 5:**
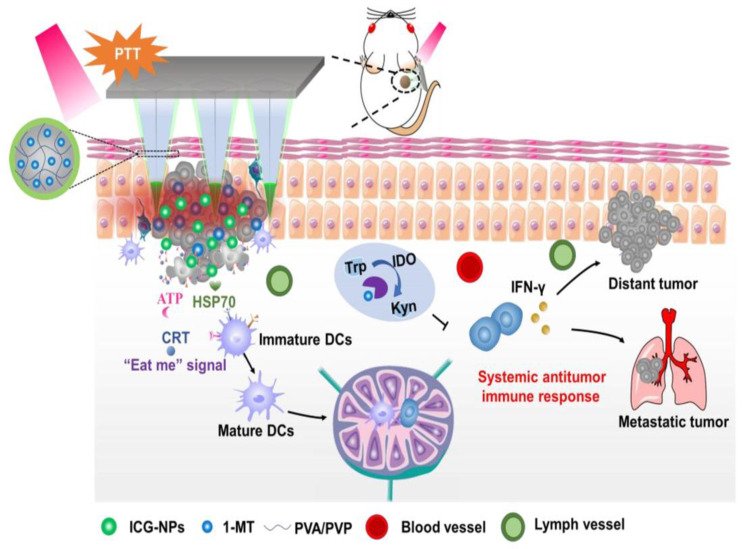
Graphical presentation of the mechanism for the antitumor effect of microneedles in combination with indocyanine green-loaded chitosan NPs (ICG-NPs).

To achieve combined synergistic effects, chemotherapeutic drugs and photothermal agents should be simultaneously delivered precisely to the tumor site. However, finding an administration route for co-delivery has remained a big challenge that can be addressed by MNs. For this purpose, DOX-encapsulated dissolvable hyaluronic acid MNs (DOX-loaded HA MNs) containing a PEGylated gold nanorod (PEGylated-GNR) as a NIR light-responsive agent was developed for human epidermoid cancer therapy [131]. In this system, PEGylated-GNR acted as a photothermal agent, and hyaluronic MNs presented a high DOX loading capacity and good skin penetration capability. It was reported that the DOX release from HA MNs can be controlled through NIR light irradiation. Furthermore, the MN-treated animals revealed a notable antitumor efficacy and the tumor growth inhibition. Moreover, several studies have applied MNs for the transcutaneous co-delivery of chemotherapeutic drugs and photothermal agents into the tumor site, indicating MNs as a promising technique for the synergistic effects of PTT and chemotherapy [77,130,132,133]. A new MNs-based system was recently introduced for PTT featuring the co-delivery of a photosensitizer (indocyanine green) loaded into chitosan NPs (ICG-NPs) and 1-methyl-tryptophan (1-MT) as an indoleamine 2, 3-dioxygenase (IDO) blockade. The ICG-NPs were concentrated on the MNs’ tip and 1-MT was encapsulated into the cross-linked PVP and PVA gel as the MNs’ core. As shown in Figure 2, ICG-NPs converted the NIR laser irradiation into heat, which could destroy the tumor cells inducing the release of tumor-associated antigens, the maturation of dendritic cells, the secretion of the immune-stimulatory cytokines, and consequently the stimulation of systematic immune responses (Figure 5). The results indicated the potential application of MN-based systems for delivering NPs, which effectively combine PTT with immunotherapy in cancer treatment [134].

After inserting MNs into the tumor site, the MNs dissolved and released ICG NPs and 1-methyl-tryptophan (1-MT) as an indoleamine 2, 3-dioxygenase (IDO) blockade. Following NIR irradiation, the PTT effect of ICG-NPs can ablate tumor cells, warn the immune system, and promote interferon-γ (IFN-γ) secretion. Furthermore, the pre-apoptotic calreticulin (CRT) translocation starts generating an “eat me” signal to the cell surface that causes the uptake of death cancer cells by APCs. Other damage-mediated molecular patterns including some heat shock proteins (HSP70) can also help in the APCs’ maturation. Then, the released 1-MT can prevent the IDO activity to catalyze the tryptophan (Trp) degradation into kynurenine (Kyn). The local co-delivery of ICG-NPs with IDO blockade not only can destroy the primary tumor cells but also inhibit the growth of distant tumor cells and lung metastases. Reprinted with permission from [134].

### 3.4. MN-Assisted NP Delivery in Photodynamic Therapy

Photodynamic therapy (PDT) is a non-invasive approach to treat various tumors and non-neoplastic diseases [135]. PDT involves combining a photosensitizer and a specific light wavelength, which can promote cytotoxic reactive oxygen species (ROS) generation at the presence of tissue oxygen resulting in the death of the damaged cells. As the systemic administration of sensitizers suffers from poor selectivity and hence skin photosensitivity, local administration is more desirable as the photosensitization only occurs at the treatment tissue [136]. Various chemical and physical methods have been investigated to improve the TDD of photosensitizers, such as lipophilic derivatization [137], chemical enhancers [138], nanoemulsions [139], sonophoresis [140], iontophoresis [141], and MNs [142]. MNs have attracted widespread attention among these methods due to their exceptional performance and low side effects.

MN-mediated PDT has been investigated for its possible applications in tumor treatment. Donnelly et al. explored the ability of solid silicon MNs for tumor therapy utilizing the poke-and-patch approach [136]. They showed that this technique enhanced the photosensitizer penetration through the murine skin. In another study, Gill et al. indicated that 5-aminolevulinic acid-coated stainless MNs (ALA-coated MNs) can enhance the TDD of the photosensitizer. Interestingly, about 57% of the tumor inhibition was achieved with only one dose of ALA (1.75 mg), while the topical cream formulation of ALA (5 mg) failed in suppressing the tumor growth [143]. The potential application of dMNs (Gantrez^®^S-97 MNs) was recently explored for the skin delivery of the NIR photosensitizer, Redaporfin™ [84]. In vitro studies showed the successful delivery of a photosensitizer as it was detected at a 5 mm depth in the skin. The in vivo biodistribution results also demonstrated the fast-initial release and localized delivery of the photosensitizer. Nevertheless, some migration away from the application site was reported by day 7, showing that widespread skin photosensitivity in patients would be improbable. In another work, the ALA tip-encapsulated fast-dissolving HA MNs (ALA@HA MNs) patch was fabricated by Zhao et al. [132]. Notably, the efficacy of PDT in xenografted tumor mice treated with ALA@HA MNs was much higher than the free ALA injection group. About 97% tumor inhibition was attained by ALA@HA MNs group (dose of 0.61 mg) while the rate was only 66% in the ALA injection group treated with a dose of 1.65 mg.

**Figure 6:**
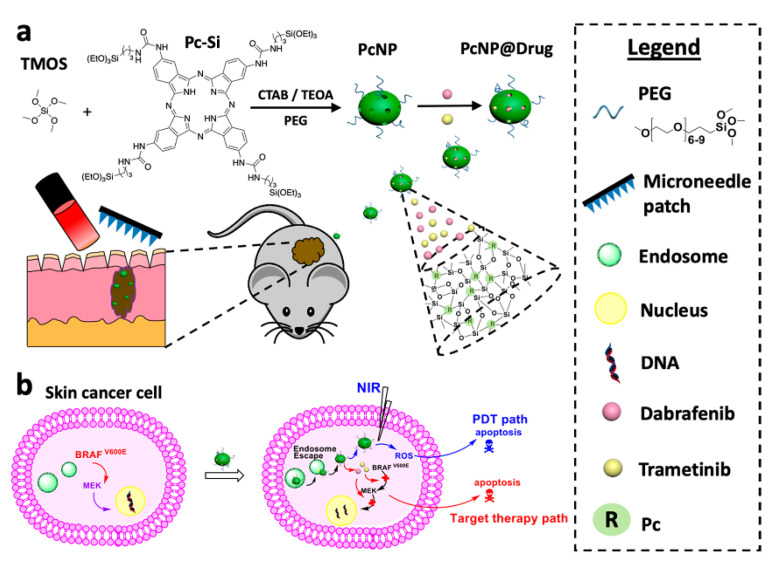
(**a**) PcNP@Drug synthesis and its skin penetration. Phthalocyanine was modified with four silicate units (Pc-Si), Pc units excited by far-red light and acting as photosensitizers, then the Pc-conjugated mesoporous organosilica nanoparticle (PcNP) was fabricated via silane co-condensation and hydrolysis using Pc-Si. In this process, hexadecyltrimethylammonium bromide (CTAB) acts as the structure-directing agent, triethanolamine (TEOA) acts as a basic catalyst and tetramethyl orthosilicate (TMOS) acts as the inorganic silica agent. To prepare PcNP@Drug, dabrafenib and trametinib, as small inhibitor drugs, were encapsulated into the PcNP pores. PcNP@Drug was then delivered to mice skin through a microneedle patch. (**b**) PcNP@Drug cellular uptake: under NIR light irradiation, PcNP@Drug produced reactive oxygen species (ROS) in-vivo and the drugs’ release of PcNP@Drug destroyed cancer cells. Reprinted with permission from [146].

As mentioned before, the delivery of highly potent photosensitizers to the tumor site is vital for the excellent therapeutic efficacy of the PDT approach. Some recent studies reported photosensitizer-loaded NP formulations for not only controlled delivery but also for combinational therapy incorporating two or more drugs [144,145]. For this purpose, Tham et al. established a mesoporous nanocarrier with a dual loading ability to encapsulate a photosensitizer and a clinically relevant therapeutic agent for combination therapy [146]. They also utilized metal MNs to promote nanocarrier penetration into the deep skin layers (Figure 6). This strategy demonstrated a synergistic killing effect on skin cancer cells and prevented the growth of the tumor cells in a 3D spheroid model in vitro. This system enhanced the skin penetration of the NPs to reach deep-seated cancer cells, which resulted in superior therapeutic efficacy through PDT in combination with the targeted therapy. Altogether, these studies suggest that the photosensitizer loaded MNs are a promising platform for enhanced and safer PDT.

When used alone, the PDT approach suffers from several challenges, such as low efficacy and systemic phototoxicity [147,148]. To address these issues, an MN-assisted delivery system was recently developed combining PDT with immunotherapy for the treatment of focal cancer. To do so, HA-MNs were incorporated with the pH-sensitive dextran NPs to deliver zinc phthalocyanine as PS and CTLA4 antibody (aCTLA4) as a checkpoint inhibitor. The in vivo study in the 4T1 mouse model showed the accumulation of zinc phthalocyanine at the tumor vicinity. PDT was first applied to kill the tumor cells, which activated the immune responses, leading to improved immunotherapy with aCTLA4. This result indicated that an MNs-assisted system in combination with NPs can be regarded as a promising co-delivery platform for the treatment of focal cancer [149].

### 3.5. MN-Assisted NP Delivery in Delivery of Therapeutic Proteins

**Figure 7:**
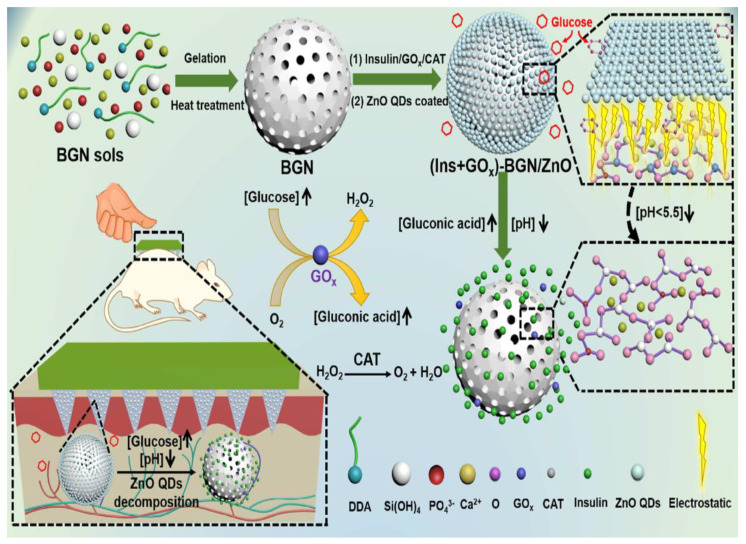
Schematic presentation of the ZnO quantum dots QDs which capped the pores of mesoporous bioactive glass NPs (MBGNs) integrated with PVP MNs for the glucose mediated TDD of insulin. After penetration into the skin and participation in the body circulation, GOx and CAT in the MBGs convert glucose to gluconic acid, resulting in decreasing the local pH and dis-assembly of ZnO QD caps, leading to the opening of MBGs’ nanopores and then releasing the insulin molecules. Reprinted with permission from [109].

MN technology has offered a revolutionary platform for protein delivery. There are generally serious challenges for the TDD of therapeutic proteins such as their susceptibility to degradation and relatively large molecule size [150]. Concerning this issue, MN technology could be an interesting delivery system capable of smoothly passing SC and delivering proteins into the systemic circulation [150]. MN technology has been widely applied in recent years for the effective delivery of proteins, including antigen [151], antibody [152], insulin [153], exendin-4 [154], and lysozyme [155]. For example, introducing MN technology to insulin delivery has been used as a non-invasive, painless, and easy-to-handle administration technique in tuning the glucose level in diabetic patients. MN-based insulin delivery systems mostly employ hollow MNs, which are currently in clinical trials [156]. As an alternative approach, dMNs were used for insulin delivery. However, the fabrication of insulin-loaded dMNs with appropriate mechanical strength and stability is a challenging task suffering from difficult skin penetration and rapid payloads release [157]. Interestingly, integrating MNs with NPs can improve mechanical properties and stability. For example, dissolving the MN composite based on PVP and insulin-encapsulated CaCO_3_ microparticles showed notable mechanical strength and sustained release in comparison with pure PVP MNs [158]. This study suggests that the combination of MNs with microparticles can lead to constant insulin release and improved therapeutic efficiency. Programmable MNs can also reduce the hypoglycemia risk associated with unnecessary drug release, thereby avoiding the side effects [159]. For this purpose, mesoporous bioactive glasses (MBGs) capped with ZnO quantum dots (ZnO QDs) were integrated with PVP dMNs exhibiting smart pH-triggered ability for the glucose-mediated TDD of insulin [109]. Glucose oxidase/catalase (GOx/CAT) were loaded as insulin and glucose-responsive factors into the MBGs’ pores that were electrostatically sealed by ZnO QDs as a pH-responsive switch. GOx/CAT content of MBGs’ pores could catalyze glucose to gluconic acid, resulting in a local pH drop leading to the dissolution of ZnO QD caps from MBGs and the insulin release from the MBGs’ pores. In vivo studies in the diabetic model showed a glucose-mediated release of insulin, along with proper blood glucose lowering action, and a lower risk of hypoglycemia (Figure 7). In another programmable MN, the surface of the insulin-loaded MBGs was modified with a glucose-sensitive layer composed of poly ethyleneimine (PEI), GOx, and CAT. Insulin release was achieved through converting the body fluids’ glucose to gluconic acid by GOx/CAT, resulting in a drop in pH and the subsequent destruction of the gatekeeping surficial layer [160]. Altogether, these studies demonstrate that the MNs-based, glucose-responsive and pH-triggered TDD can be a successful strategy in the diabetes treatment.

The ongoing development of smart insulin delivery based on MN technology in combination with NPs has shown the potential to improve the quality of life of diabetic patients. In recent works, the MNs integrated with insulin-loaded, H_2_O_2_-responsive NPs were designed to obtain fast and painless administration [161,162]. In this system, GO_x_ was co-loaded into the NPs to catalyze blood glucose to gluconic acid and generate H_2_O_2_ as a reaction byproduct resulting in NPs disintegration and insulin release. Therefore, the NPs act as both the glucose-sensing agent moieties and the insulin release actuator. NPs can provide basal insulin release in a controllable manner. Moreover, they facilitate insulin release in response to a hyperglycemic condition [163]. These results indicate that stimuli-responsive glucose-mediated TDDSs based on MNs in combination with NPs have potential applications in diabetes management.

MNs have been utilized for transdermal vaccination and the immunotherapy of skin tumors. Zhou et al. developed MNs containing transfersomes co-encapsulated anti-PD1 (antigen) and polyinosinic:polycytidylic acid (adjuvant) which were functionalized with αCD40 (DCs targeting ligand). The accumulation of the transferosomes in tumor-draining lymph nodes enhanced the maturation of DCs and improved Th1 immune responses. An improved T cell activation and infiltration was reported in a mouse melanoma model. Moreover, the activity of regulatory T cells decreased in the tumor site, reverting the immunosuppressive tumor microenvironment [164]. This study indicated the potential application of MNs in combination with transfersomes as a promising platform for cancer immunotherapy. In another study, MicronJet600 hollow MNs were used to deliver an auto-antigen peptide conjugated with AuNPs. In vitro investigations showed AuNPs uptake by DCs and the activation of naïve T cells. AuNPs can also facilitate the temporal and spatial delivery of peptides. Therefore, AuNP–peptide formulations, which are currently under clinical investigation, can be incorporated into MNs for immunotherapy applications [165].

### 3.6. MN-Assisted NP Delivery in Vaccine Delivery

Most vaccines are currently administered using hypodermic needles, necessitating expert administration, cold chain storage, and the transportation of liquid formulations. In addition, most vaccines are formulated in liquid necessitating close temperature control during transport, storage, and distribution (i.e., cold chain) [166]. The issues of poor vaccine transport through the skin barrier, patient compliance, and cold chain can be addressed by MNs. MNs can painlessly bore the SC and canalize the epidermis to improve the vaccination as well as dependency on the cold chain and the need for reconstitution [167]. Additionally, MNs should be regarded as a unique strategy for the delivery of antigen to immune cells such as DCs within the skin, which is an essential problem in vaccine delivery. To date, MN reports have demonstrated similar or even higher immunogenicity and dose sparing [168]. Several investigations have reported the application of different MN strategies and their use in various vaccine formulations, including influenza, and Human papillomavirus (HPV). For example, inactivated influenza virus vaccine was encapsulated in dMNs, and sucrose or trehalose was used to stabilize the antigen. The results indicated that the stabilization and vaccine drying through lyophilization can lead to greater vaccine stability and in vivo immunogenicity compared with the conventional vaccine preserved for 1 month at 45 °C [169]. Accordingly, dMNs can reduce the dependency on the cold chain, improve the thermostability, eliminate the need for reconstitution, and simplifying vaccine distribution. For more detailed information regarding MNs-based vaccination systems, please refer to several excellent reviews [25,170,171].

As mentioned earlier, MNs have contributed to the delivery of many NPs into the skin that can be extended to vaccine delivery. The main reason behind the use of NPs in combination with MNs to deliver the antigen is the improved stability and controlled release of antigen for inducing higher immunogenicity. For example, chicken OVA was encapsulated into PLGA-NPs, which was then incorporated in dMNs. Using this approach, MNs slowly released the antigen to lymph nodes occupied with DCs. This strategy led to the successful in vivo immune system activation against influenza and melanoma tumors [126]. In another study, monophosphoryl lipid A, OVA, imiquimod, and Toll-like receptor (TLR) agonists were encapsulated in PLGA NPs, which were then intradermally delivered through hollow MNs. Unlike intramuscular injection, the MNs generated higher levels of IgG2a antibody and IFN-γ-producing lymphocyte [172]. Guangsheng et al. compared different types of nanocarriers to modulate the immune response by hollow MNs. To do so, OVA was loaded into MSNs, liposomes, PLGA, or gelatin NPs with or without polyinosinic:polycytidylic acid as an immunostimulant. Liposome and PLGA induced significantly higher IgG2a response. Moreover, liposomes led to CD4^+^ and CD8^+^ T cell activation [173]. Table 3 summarizes the studies on the MNs’ application in the transcutaneous delivery of antigen-loaded NPs.

### 3.7. MN-Assisted NP Delivery for Gene Therapy

Gene therapy, or replacing a malfunctioning gene with therapeutic nucleic acids [192], can be applied in the treatment of a wide variety of genetic skin disorders, cutaneous cancers, and wound healing. Transdermal DNA delivery has several advantages, including localized delivery, large surface area, and evading first-pass metabolism [193]. However, the SC limits the TDD of macromolecules, particularly nucleic acids. To date, several physical strategies, such as the gene gun, iontophoresis, sonophoresis, needle-free liquid jet injections intradermal injection, electroporation, and MNs have been employed to deliver DNA therapeutics across the skin [193]. The application of MNs in gene delivery has been explored with different delivery approaches, particularly with solid MN arrays, which have been shown for bare DNA or its combination with NPs [71,194]. For the first time, the bare plasmid DNA (pDNA) encoding firefly luciferase was applied topically to introduce DNA into the disrupted skin of the shaved dorsum of BALB/c mice by blunt-tipped silicon MNs. The results showed a 2800-fold increase in gene expression when the solid MN was used in scraping the SC barrier instead of a topical application. In another study, the pDNA encoding the hepatitis B surface antigen induced a robust and high antibody titer via silicon MNs compared to intradermal injection. Furthermore, the combinatorial use of NPs enables us to appropriately control the release kinetics, gene loading, and cellular uptake of genetic materials delivered by MNs. Ruan et al. developed MNs coated with BRAF siRNA (siBraf) complexes with cell-penetrating peptide octaarginine (R8) for melanoma treatment. In vivo experiments exhibited the significant inhibition of melanoma development, apoptosis, and the suppression of cell proliferation in mice bearing melanoma [195].

MNs are especially considered in the transcutaneous delivery of DNA vaccines. For example, the double conjugate of PEI to mannose (Man) and cell-penetrating peptide (CPP: a sequence of TAT: RRRQRRKKRC-SH) were used to condense the DNA vaccine, which was then applied in transcutaneous immunization using solid MNs. This system effectively activated the Trp2-specific response, leading to effective immune system activation against B16 melanoma in BALB/c mice [128]. In this same research performed by Xu et al., MNs were applied co-delivery of DNA vaccine and low-dose paclitaxel (PTX) encapsulated in polymeric nanocomplex for cancer immunotherapy. In this system, polymeric nanocomplex was constructed using low-dose PTX as a neoadjuvant that was physically loaded in sulfobutylether-cyclodextrin (SBE), and the PTX/SBE further acted as an anionic crosslinker to self-assemble with the cationic mannosylated *N*, *N*, *N*-trimethyl chitosan/DNA polyplexes. MNs assisted in the co-delivery of the DNA vaccine and PTX to DCs, which resulted in synergistic effects on the DCs maturation and antigen-presenting function, consequently enhancing the immune stimulation and decreasing the immune escape [196].

## 4. Advances in Clinical Trials and Commercialization of MNs

As mentioned earlier, the applicability of MNs in TDD has been investigated since 1998 [26]. To date, numerous pre-clinical and clinical trials have been conducted to examine the safety and efficacy of MNs for cosmeceutical, diagnosis, and therapeutic applications [18]. As presented in Table 4 and Table 5, advances in MN fabrication technology have led to the commercialization of MNs for clinical applications. During the last decade, clinical trials have been aimed at using MNs for vaccination and insulin delivery. The results from the clinical trials on cosmeceutical applications have shown that MN rollers, as a type of solid MNs, are the most attractive choice. According to Table 4, the application of MNs combined with NPs for TDD is limited to the proof-of-concept pre-clinical studies and there is only one report of a phase 1 clinical trial of the intradermal administration of human C19A3 proinsulin peptide coupled to gold NPs using MNs in type 1 diabetes [197]. Due to the significant features of NPs and their promising clinical outcomes, it is expected that a combination of NPs’ and MNs’ application grow fast in the future, but further studies are required to exploit the therapeutic and diagnostic potentials of smart MNs.

## 5. Current Challenges and Future Perspectives

Given the thorough research performed in recent years on various types of MNs, there are several critical issues to address for their widespread applications in clinical settings. MN applications may lead to short- and long-term safety concerns in patients. It is generally believed that there is no major complication with the short-term use of MNs; nevertheless, the long-term application of MNs can cause erythema (skin redness) as well as pain based on their size and the number of needles. In addition, the effectiveness of MNs as a pore-forming pretreatment depends on the availability of their open micropores. The skin type can also affect the micropore closure time following the MN’s application. Recently, it was reported that the micropore closure time can vary in human subjects of different ethnic/racial backgrounds, with a longer micropore closure time in colored skin. These findings indicate that further research is warranted to verify the safety and efficacy of MNs in a variety of human populations [199]. Another issue is the large-scale production which may hinder their clinical translation. There are also some other limitations including (i) a lack of specific regulatory guidelines for quality control tests of MNs (e.g. geometry, mechanical strength, and in vitro/in vivo correlation), (ii) human safety requirements for the clinical use of MNs, (iii) pharmacokinetic and pharmacodynamic evaluations of MNs are vital to confirm their safety and efficacy, (iv) manufacturing sterile MNs in aseptic conditions or using final sterilization processes are expected to significantly increase production costs, (v) specialized industrial machinery, several fabrication steps, particularly for the coated MNs, and clean room facility requirements upfront investment, and (vi) preserving MNs against moisture and microbial contamination through using suitable packaging [24]. Hence, the complex and costly production of MNs as well as several application-related concerns can hinder their clinical translation. New fabrication methods especially micromachining and 3D printing technologies are expected to reduce the cost and fabrication steps in the future.

Due to the emerging features of NPs and their promising clinical outcomes, rapid developments in NP-laden MNs are expected, but further research is required to exploit their therapeutic and diagnostic potentials. Apart from the efficacy of NPs, the safety of their use via the transdermal route should be addressed. Furthermore, incorporating NPs into the MN matrix can change their mechanical strength. There are a variety of modes resulting in the MNs’ mechanical failure, such as base-plate fracturing, MNs folding, and warping. Consequently, the mechanical properties of MNs should be characterized using different mechanical tests including axial and transverse force, base-plate strength, and shear strength analysis [24]. Moreover, it may change the NPs stability and dispersibility which can deteriorate their function. In addition, more investigations are still warranted to elucidate the therapeutic action of NPs in combination with MNs because most studies have only reported pharmacological and clinical outcomes in animal models without necessarily addressing the molecular basis of their observation. There is also no reliable preclinical model to precisely investigate the pharmacokinetics and biodistribution of NPs when applied using MNs, hindering the clinical translation of these systems. Last but not the least, the aseptic, large scale production, and appropriate storage conditions should be concerned.

## 6. Conclusions

Minimally invasive TDD has remained a challenge requiring more advanced delivery systems to address the limitations associated with conventional therapies. Among numerous TDD systems, NPs have attracted significant attention in recent years due to their potential features such as effective delivery carriers, not only to reduce the side effects associated with conventional formulations, but also to enhance skin permeation, depot drug action, and targeted delivery to impaired skin cells. Moreover, due to the interesting characteristics of certain NPs, including optical, photothermal, and superparamagnetic properties, novel therapeutic, imaging, and biosensing applications are emerging. MNs-based delivery systems have attracted much attention in improving the function of NPs in TDD due to their non-invasive and pain-free delivery characteristics, without a first-pass effect as well as infection and safety problems. Interestingly, the MNs-array technology can shuttle NPs directly into the skin layers. In recent years, MNs, in combination with NPs containing therapeutic agents, have advanced TDD to a new level to effectively treat skin disorders. A combination of MNs and NPs has shown numerous benefits over conventional systems, such as the improved skin penetration of NPs, prolonged or controlled drug release, and the possibility of new add-on therapeutics (e.g., PTT and PDT). Although not fully explored, NPs might play a vital role in cancer chemotherapy using MNs. Moreover, these combined systems have been especially explored for vaccination, immunotherapy, and gene delivery. For a successful delivery of NPs in combination with MNs, several features are warranted, such as the type, physicochemical, pharmacokinetic, and biologic features of NPs as well as the strategy used for delivery by MNs. Overall, MNs can be applied in the TDD of NPs, either drug-loaded or alone, by properly developing and reliably evaluating their practical aspects in clinical trials. These combined systems would have a significant impact on the future of nanomedicine to effectively treat skin disorders as well as systemic applications.

## Figures and Tables

**Table 1:** Advantages, disadvantages, and applications used for different types of microneedles (MNs).

Type of MNs	Advantages	Drawbacks	Applications
Solid	Technically simple, without any pump or loading/coating procedure, small doses can be administered	Two-step administration procedure, no exact dosing, drugs need to be reformulated	Skin pretreatment for the delivery of insulin [55], cosmetics [56], vaccines [57], potassium chloride [58], monitoring of lactate and glucose [59], urea sensing [60]
Coated	MN strength is retained after coating, without any patch or pump, precise dosing is possible	Appropriate coating technique is needed, limited to small doses, drugs need to be reformulated, coatings might lessen MN sharpness	Delivery of vaccines [61], insulin [62], proteins [63], desmopressin [64], parathyroid hormone [65], sampling, isolation and identification of biomarkers [66]
Hollow	Drug delivery rates can be controlled, delivery of a relatively high liquid volume is plausible, possible combination with lab-on-a-chip devices, precise dosing	Possible risk of clogging, reduced MN strength, possible risk of drug leakage, complex devices	Delivery of vaccines [67], insulin [68], cell therapy [69], delivery of mRNA [70], DNA (pDNA) [71], biofluid extraction and bio-signal detection [72,73], colorimetric detection of glucose [74]
Dissolving	No need for any pump or patch, precise dosing is possible, no sharp waste, low preparation costs	Small drug doses may be lost throughout the encapsulation/absorption procedure, low strength, low penetration ability, limited to small drug doses, drug reformulation is needed	Delivery of vaccines [75,76], insulin [77], therapeutic peptides [78], triamcinolone acetonide [79], doxorubicin [80], epidermal growth factor and ascorbic acid [81], adenosine [82], vitamin B12 [83], near-IR photosensitizer (Redaporfin™) [84], sodium nitroprusside in combination with sodium thiosulfate [85], DNA extraction [86]
Hydrogel-forming	No need for any pump or patch, precise dosing is possible, no sharp waste	Small drug doses may be lost throughout the encapsulation/absorption procedure, limited to small drug doses, low strength, and penetration ability, drug reformulation is needed	Delivery of vaccines [87], metformin hydrochloride [50], methotrexate [53], caffeine [46]. glucose-responsive insulin delivery [51], stimulus-responsive ibuprofen delivery [47], lithium monitoring [88], theophylline, caffeine and glucose monitoring [89]

**Table 2:** Microneedle-assisted transdermal delivery of nanoparticles (NPs).

Type of MNs	Carrier	Drug	Outcome	Ref.
Solid (stainless steel)	Microemulsions	Propranolol	Enhanced permeation	[103]
Solid (silicon)	Dextran NPs	Rosiglitazone CL 316243	Localized and painless administration in a safe and effective manner	[104]
Solid (silicon)	Microemulsion	Tetramethyl pyrazine	Enhanced percutaneous absorption of drug-loaded microemulsions by MNs	[68]
Solid (rolling MNs)	Ethosomes	Paeoniflorin	Enhanced skin penetration, no synergistic effect in combination of MNs with ethosomes	[101]
Solid (not defined)	NLC	Alkaloids	Enhanced skin penetration with combination of MNs and NLCs, increased transdermal bioavailability of alkaloids, consistent blood drug concentrations	[105]
Solid (stainless steel)	Polymeric NPs (carboxymethyl chitosan)	5-Fuorouracil	Localized and painless administration, low side effects	[106]
Dissolving (PVP)	PLGA hollow microspheres	Alexa 488 and Cy5 (model compounds)	Co-delivery into the skin	[43]
Dissolving (PEG-MGQD)	nanocomposites of chitosan and magnetic graphene quantum dot	Lidocaine hydrochloride	Delivery of small and large molecule therapeutics	[107]
Dissolving (PVP)	PLGA nano/microparticles	Vitamin D3	Delivery to deep skin layers and sustained release	[108]
Dissolving (PVP)	Hollow mesoporous silica nanocomposites	Metformin	NIR-triggered TDD and photothermal-responsive delivery	[109]
Dissolving (PVP)	PLGA@chitosan and PCL@chitosan NPs	Doxycycline	Enhanced retention and improved dermatokinetic profile of doxycycline	[110]
Dissolving (PVP)	Nanosuspension	Doxycycline, Albendazole, and Ivermectin	Enhance retention in the dermis	[111]
Dissolving (PVA/PVP)	PCL NPs	Carvacrol	Enhanced skin retention, sustained therapeutic effect	[112]
Dissolving (HA)	HA NPs	Rhodamine B	Enhanced skin penetration, sustained release	[113]
Dissolving (PVA)	Nanosuspension	Curcumin	Improved intradermal delivery	[114]
Dissolving (HA)	MPEG-PCL NPs	5-Fuorouracil and indocyanine green	NIR-responsive delivery, synergistic chemo-photothermal effect	[115]
Dissolving (HA)	Micelles	Curcumin	Enhanced transdermal permeation	[116]
Dissolving (PVA/PVP)	SLNs	Doxycycline, Diethylcarbamazine, and Albendazole	Enhanced retention in the dermis layer, high bioavailability, accumulation in lymph nodes	[117]
Dissolving (PVA)	Micelles	Rhodamine B	TDD of kidney targeting NPs	[118]
Hydrogel-forming (PLGA)	Hydrogel microparticles	Rhodamine B	Sustained release	[119]

PCL: poly (ε-caprolactone); MPEG: monomethoxy-poly (ethylene glycol).

**Table 3:** Vaccine delivery studies with different MNs regarding the type of vaccine and NPs.

Type of MNs	Vaccine Type	Carrier	Outcome	Ref.
Solid	Fluvax vaccine + CXCL1-specific siRNA	Liposome	Effective silencing of CXCL1 gene in skin	[174]
Solid	HPV-16 E6/E7 DNA	PVP	More potent vaccination than intramuscular administration, delayed TC-1 tumor growth	[175]
Solid	Ovalbumin	Hyaluronan	Humoral and mucosal immune activation, strong immune-recall responses, strong immunization	[176]
Solid	Tetanus toxoid	Chitosan	Higher IgG2a level in comparison to commercial vaccine, balanced Th1/Th2 ratio	[177,178]
Coated	HIV-1 p24 Gag peptide	Polypropylene sulfide	Efficient uptake without adjuvant, potent HIV-1specific CD4^+^ T-cell responses	[179]
Coated	pH1N1 DNA	Polyplex containing PLGA/poly ethyleneimine (PEI)	Rapid dissolution of polyplex which induced potent humoral immune response	[180]
Hollow	H1N1 virus	Virus like nanoparticles (VLPs)	New vaccination approach	[181]
Hollow	Ovalbumin	PLGA	Protective T cell-mediated immunity	[182]
Hollow	Plasmid DNA	Niosome	High level of IgG titer	[183]
Hollow	Diphtheria toxoid	Liposome	High level of IgG2a	[184]
Hollow	Peptide/ Human papillomavirus	Liposome	Strong T helper responses	[184]
Dissolving	Ovalbumin	Liposome	High IgG level, enhanced immune response	[185]
Dissolving	Ovalbumin	Liposome	Effective mucosal immunization via oral route vaccination	[186]
Dissolving	pEGFP-N1 plasmid DNA	PVP and PVA	Superior DNA preservation by PVA than PVP	[187]
Dissolving	Ovalbumin	Liposome	Enhanced immune response, balanced Th1 and Th2 humoral immune responses	[188]
Dissolving	Ovalbumin	Transfersomes	Increased IgG2a/IgG1 ratio, specific Th1 antigen-specific immunizations Immune response in lymph nodes	[189]
Dissolving	Hepatitis B virus	Liposome	Strong cell-mediated immune response HBV, CD8^+^ T cells population increase significantly	[186]
Dissolving	Ovalbumin Plasmid	OSM-(PEG-PAEU) *	Humoral and cellular immunity, activation of cytotoxic CD8^+^ T cells	[190]
Dissolving	DNA	Pluronic P123/PEI	Higher humoral and cellular immunity compared to IM administration	[177,178]
Dissolving	SIINFEKL peptide	Cubosome	Efficient local delivery.	[191]

* Oligo sulfamethazine conjugated poly (β-amino ester urethane).

**Table 4:** Clinical trials and their current status [198].

Field	Title	Type of MNs	Condition/Disease	Phase	Status	Clinical Trial Registry Number
Therapeutic	2010/2011 trivalent influenza vaccination	Solid (MicronJet)	Influenza	Not provided	Completed	NCT01304563
	The effect of microneedle pretreatment on topical anesthesia	Solid (MN roller)	Pain	Not provided	Completed	NCT02596750
	The use of microneedles to expedite treatment time in photodynamic therapy	Solid (MN roller)	Keratosis, actinic	Not provided	Completed	NCT02594644
	The use of microneedles with topical botulinum toxin for treatment of palmar hyperhidrosis	Solid (Sham MN)	Hyperhidrosis	I	Completed	NCT03203174
	A study to determine the patient preference between Zosano Pharma parathyroid hormone (ZP-PTH) patch and Forteo pen	Coated titanium (ZP-PTH MN patch)	Postmenopausal Osteoporosis	I	Completed	NCT02478879
	Phase 2 study of BA058 (Abaloparatide) transdermal delivery in postmenopausal women with osteoporosis	Coated 3 M microstructured transdermal system	Post-menopausal osteoporosis	II	Completed	NCT01674621
	Dose sparing intradermal H1N1 influenza vaccination device	Hollow (MicronJet 600™)	Influenza infection	Not provided	Completed	NCT01049490
	Immunogenicity of inactivated and live polio vaccines	Hollow (MicronJet 600™)	Poliomyelitis	III	Unknown	NCT01813604
	Safety study of suprachoroidal triamcinolone acetonide via microneedle to treat uveitis	Hollow	Uveitis	I/II	Completed	NCT01789320
	Routes of immunization and flu immune responses	MN injection (not defined)	Influenza	I/II	Completed	NCT01707602
	Comparison of 4 influenza vaccines in seniors	Hollow (Becton Dickinson (BD) MN)	Influenza	IV	Completed	NCT01368796
	Immunogenicity of the inactivated split-virion influenza vaccine in renal transplant subjects	Hollow (BD Soluvia™)	Influenza Orthomyx-oviridae infection	II	Completed	NCT00606359
	Atopic dermatitis research network (adrn) influenza vaccine study	Pre-filled or hollow (Fluzone^®^)	Dermatitis, atopic	Not provided	Completed	NCT01737710
	Immunogenicity study of the influenza vaccine in adults	Hollow (BD MN injection)	Orthomyx-oviridae infection Influenza	II	Completed	NCT00258934
	Study of inactivated, split-virion influenza vaccine compared with the reference vaccine Vaxigrip^®^ in the elderly	Hollow (BD MN injection)	Orthomyx-oviridae infection Influenza Myxovirus infection	III	Completed	NCT00383526
	Intradermal versus intramuscular polio vaccine booster in HIV-infected subjects	Hollow (MicronJet 600™)	Polio immunity	II	Completed	NCT01686503
	Varicella zoster virus (VZV) vaccine for hematopoietic stem cell transplantation	Hollow (MN syringe)	Varicella zoster infection	II/III	Completed	NCT02329457
	Insulin delivery using microneedles in type 1 diabetes	Hollow (glass)	Type 1 Diabetes Mellitus	II/III	Completed	NCT00837512
	A pilot study to assess the safety, pharmacokinetics/ pharmacodynamics (PK/PD) of insulin injected via MicronJet™ or conventional needle	Hollow (MicronJet™)	Intradermal injections	Early phase I	Completed	NCT00602914
	Pharmacokinetic comparison of intradermal versus sub-cutaneous insulin and glucagon delivery in type 1 diabetes	Hollow (MicronJet™)	Type 1 Diabetes	II	Unknown	NCT01684956
	Multi-day (3) in-patient evaluation of intradermal versus subcutaneous basal and bolus insulin infusion	Hollow (BD Research Catheter)	Diabetes	I/II	Completed	NCT01557907
	Safety and efficacy of ZP-glucagon to injectable glucagon for hypoglycemia	Not defined	Hypoglycemia	I	Completed	NCT02459938
	Study on the effects on blood glucose following intradermal and subcutaneous dosing of insulin in diabetic patients	Hollow (BD Research Catheter)	Diabetes	I/II	Completed	NCT01120444
	Pharmacokinetics/dynamics of basal (continuous) insulin infusion administered either intradermally or subcutaneously	Hollow (BD Research Catheter)	Diabetes Mellitus, Type 1/2	I/II	Completed	NCT01061216
	A study to assess the safety and efficacy of a microneedle device for local anesthesia	Hollow (MicronJet™)	Local anesthesia intradermal injections	Not provided	Completed	NCT00539084
	Safety demonstration of microneedle insertion	Gold/silver coated or uncoated hollow MNs	Allergic reaction to nickel	NA (safety demonstration)	Completed	NCT02995057
	Microneedle patch study in healthy infants/young children	Dissolvable	Vaccination skin absorption	Not provided	Completed	NCT03207763
	Microneedle array–doxorubicin (MNA-D) in patients with cutaneous T-cell lymphoma (CTCL)	Dissolvable	Cutaneous T cell lymphoma	I	Recruiting	NCT02192021
	Inactivated influenza vaccine delivered by microneedle patch or by hypodermic needle	Dissolvable	Influenza	I	Completed	NCT02438423
	Microneedle patch for psoriatic plaques	Dissolvable (MN-HA patch)	Psoriasis	Not provided	Unknown	NCT02955576
	Safety and efficacy of ZP-zolmitriptan intracutaneous microneedle systems for the acute treatment of migraine	MN patch (not defined)	Acute migraine	II/III	Completed	NCT02745392
Cosmetic	Microneedling plus the universal peel for acne scarring	Solid (MicroPen)	Acne scarring	Not provided	Completed	NCT02174393
	Comparison of efficacy between fractional microneedling radiofrequency and bipolar radiofrequency for acne scar	Solid (Microneedling radiofrequency device)	Acne scarring	Not provided	Completed	NCT02207738
	Comparison of treatments for atrophic acne scars	Solid (Dermaroller)	Acne scarring	Not provided	Unknown	NCT02025088
	Comparison of the efficacy of micro-holes versus laser-assisted dermabrasion, for repigmenting in vitiligo skin	Solid (Dermaroller)	Vitiligo-macular depigmentation	Not provided	Unknown	NCT02660320
	Transplantation of Basal Cell Layer Suspension Using Derma-rolling System in Vitiligo	Solid (Dermaroller)	Vitiligo	Not provided	Unknown	NCT02962180
	Safety and efficacy of the EndyMed Pro system using RF microneedles fractional skin remodeling	Solid (EndyMed Pro™ RF Microneedles)	Aging	Not provided	Unknown	NCT02368626
	Evaluating the efficacy of microneedling in the treatment of androgenetic alopecia	Solid (Microneedling)	Androgenetic alopecia	I	Unknown	NCT02154503
	Performance of the ePrime System for Cellulite	Solid (ePrime Syneron Candela)	Cellulite	Not provided	Unknown	NCT02489994
	Tolerability study of the application of a 3M microstructure transdermal system	Solid (Transdermal Microchannel Skin System)	Healthy	I	Completed	NCT01257763
	Teosyal^®^ PureSense redensity [I] injection using Micronjet^®^ needle in the treatment of crow’s feet wrinkles	Hollow (MicronJet™)	Crow’s feet wrinkles	IV	Completed	NCT02497846
Diagnostic	Physiological study to determine the allergic skin activity after different skin preparation	Solid (Micro Skin System, 3M)	Birch pollen allergy	I	Completed	NCT01628484
	Minimally Invasive Sensing of Beta-lactam Antibiotics (MISBL)	Solid	Healthy volunteers	I	Completed	NCT03847610
	Analysis of non-invasively collected microneedle device samples from mild plaque psoriasis for use in transcriptomics profiling	Solid MNs as sampling device	Psoriasis vulgaris	Cohort, prospective	Completed	NCT03795402
	Optimization of tuberculosis intradermal skin test	Hollow (BD Research Catheter)	Healthy volunteers	Not provided	Completed	NCT01611844
	Glucose measurement using microneedle patches	Hydrogel forming	Diabetes (diagnostic)	Not provided	Completed	NCT02682056

Unknown: study has passed its completion date and status has not been verified in more than two years.

**Table 5:** Commercially available MNs.

Type of MNs	Product Name	Company Name	Description of the Product	Use
Solid	Dermapen	Dermaroller GmbH	An array of 12 needles loaded on an electric motor unit fitted with a spring that punches skin (412–700 cycle/min)	Treatment of isolated scars, skin lesions and wrinkles, appropriate for smaller areas of the skin
	Dermaroller	Whitelotusbeauty	Contains 192 titanium needles (0.5 mm long) in cylindrical assembly	Cosmetic application and skin care with cream and serum, appropriate for larger areas of the skin
	Dermastamp	Whitelotusbeauty	An array of 40 needles loaded on an electric motor unit with controlled motion back and forth like stamp	Collagen induction therapy for skin scars, age spots, varicella scars and wrinkles, appropriate for smaller areas of the skin
	DermaFrac	Dermafrac.co	Very small stainless steel MNs roller equipped with electric power source and instrument for serum infusion, also contains light emitting devices	Wrinkles, skin ageing, hyperpigmentation, acne, uneven skin tone
	Onvax	Becton Dickinson	An array of silicon or plastic micro-projections on a hand-held applicator	Vaccine delivery
	LiteClear	Nanomed skincare	Silicon MNs pen for skin pretreatment	Treatment of acne and skin blemishes
	h-patch	Valeritas	Small adhesive MNs, hydrolytically regulated	Basal and bolus delivery of insulin
	Beauty Mouse	Dermaroller GmbH	Three rollers of 50 mm width and a total of 480 MNs, creating fine microchannels for enhanced penetration	Increasing the skin’s sensitivity towards anti-cellulite creams
	NanoCare	NanoPass Inc.	A small hand-held device for the rejuvenation of skin and to boost the cosmetic effect of topical applications	Cosmetic
	Adminstamp	AdminMed	MNs array attached to the applicator with six stainless steel screws (1400 µm-long MNs on 1 cm^2^ circular array), compatible with all sterilization methods	Transdermal drug delivery through the skin with excellent skin sensation and cosmetics
Coated	MacroFLUX™	Zosano pharma	PTH-coated titanium MN patch	Osteoporosis
	Nanopatch	Vaxxas	Silicon patch (1 cm^2^) made up thousands of coated micro-projections	Polio vaccine
Hollow	3M MTS	3M Corp	MN patch containing 351 needles/cm^2^ (650 µm length)	Skin treatment before dermatological application
	FLUARIX	GlaxoSmithKline Biologicals	Three MNs (600 µm length) attached to a syringe	Influenza vaccine delivery
	Micronjet	Nanopass	Four silicon MNs (450µm length) attached to the tip of a plastic adapter	Intradermal vaccine delivery
	Fluzone	Sanofi Pasteur Inc Becton Dickinson	A hand syringe gun unit that injects 1.5 mL vaccine solution by a micro-injector in intradermal site by a 1.5 mm MN tip	Influenza vaccine
	Soluvia	Sanofi-Aventis Becton Dickinson	MNs (length 1.5 mm) for intradermal delivery	Influenza vaccine
	Microinfusor	Becton Dickinson	An automated hands-free system consists of an electrical pump connected to a hollow MN patch (capacity 0.2–15 mL)	Influenza vaccine, insulin, and highly viscose biotech drug
	Nanoject	Debiotech	Based on MEMS technology, hollow MN patch (length 300 to 1000 µm), featuring side holes which prevent sticking in the MN channels during skin penetration	Intradermal drug delivery and injection of diagnostic fluid
	Micro-Trans	Valeritas Inc.	Hollow MNs constructed with metal or biodegradable polymers	Intradermal drug delivery
	Intanza/IDflu	Sanofi-Aventis Becton Dickinson	MNs (length 1.5 mm) combined with a needle shielding system and a 0.1 mL injection volume	Influenza vaccine
	AdminPen	AdminMed	Forty-three needles (length 1100 µm) on 1 cm^2^ circular microneedle array, stainless steel liquid injector device attached to a standard syringe	Liquid formulations (vaccines, drug)
	Microinject	Nanopass	Four hollow silicon MNs (length 250 µm), fitted with a syringe for intradermal injection	Influenza vaccine
	DebioJect	Debiotech	One or several hollow silicon MNs with a length ranging from 350 to 900 µm and a side protected delivery holes, injections up to 0.5 mL in a few seconds	Vaccine delivery
Dissolving	Drugmat^®^	Theraject Inc.	Sumatriptan-loaded dMN patch made from a sugar polysaccharide	Migraine
	Vaxmat^®^	Theraject Inc.	Sumatriptan-load dMNs	Migraine
	MicroCor	Coriumintl	PTH-loaded dissolvable peptide MN patch	Osteoporosis
	MicroHyala	CosMed	MN patch made of biocompatible hyaluronic acid	Wrinkle treatment, influenza vaccine

PTH: parathyroid hormone, microstructured transdermal system technology.

## Data Availability

Not applicable.

## Abbreviations

5-Aminolevulinic acid (ALA); aminolevulinic acid (ALA)-coated stainless MNs (ALA-coated MNs); antibody-dependent cell-mediated cytotoxicity (ADCC); antigen-presenting cells (APCs); anti-the programmed death-1 (aPD1); bubble MNs (BMNs); carbon nanotubes (CNTs); carbon nanofibers (CNFs); cell-penetrating peptide (CPP); dissolvable MNs (dMNs); DOX-loaded dissolvable hyaluronic acid MNs (DOX-loaded HA MNs); graphene oxide (GO); glucose oxidase/catalase, (GOx/CAT); gold NPs (AuNPs); hafnium oxide (HfO_2_); hexadecyltrimethylammonium bromide (CTAB); hydrogen peroxide (H_2_O_2_); hyaluronic acid (HA); hydroxyethyl methacrylate (HEMA); dendritic cells (DCs); ethylene glycol dimethacrylate (EGDMA); hydroxyethyl methacrylate (HEMA); indocyanine green loaded into chitosan NPs (ICG-NPs); indoleamine 2,3-dioxygenase (IDO); Langerhans cells (LCs); magnetic graphene quantum dot (MGQD); magnetic resonance imaging (MRI); mannose (Man); mesoporous bioactive glasses (MBGs); mesoporous silica nanoparticles (MSNs); 1-methyl-tryptophan (1-MT); microneedle arrays (MNs); microneedles (MNs); monomethoxy-poly (ethylene glycol) (MPEG); multivesicular vesicles (MVV); nanocarriers (NCs); nanoparticles (NPs); nanostructured lipid carriers (NLCs); near-infrared (NIR); oil-in-water (O/W); oligo sulfamethazine conjugated poly (β-amino ester urethane (OSM-(PEG-PAEU)); organosilica nanoparticle (PcNP); ovalbumin (OVA); paclitaxel (PTX); PEGylated gold nanorod (PEGylated-GNR); photodynamic therapy (PDT); photodynamic therapy (PDT); photothermal ablation therapy (PTT); photothermal therapy (PTT); phthalocyanine (Pc); plasmid DNA (pDNA); siRNA (siBraf); stratum corneum (SC); octaarginine (R8); poly(ε-caprolactone) (PCL); polyethyleneimine (PEI); poly methyl vinyl ether and maleic anhydride (PMVE/MA); polyethylene glycols (PEGs); poly-lactic-acid (L-PLA); polylactic-co-glycolic acid (PLGA); polyvinylpyrrolidone (PVP); quantum dots (QDs); reactive oxygen species (ROS); silver nanoparticles (AgNPs); transdermal drug delivery systems (TDDSs); tumor-draining lymph nodes (tdLN); zinc oxide (ZnO); scanning electron microscopy (SEM), solid lipid nanoparticles (SLNs); stratum corneum (SC); sulfobutylether-cyclodextrin (SBE); superparamagnetic iron oxide (SPIONs); surface plasmon resonance (SPR); surface-enhanced Raman scattering (SERS); tetramethyl orthosilicate (TMOS); titanium dioxide (TiO_2_); transdermal delivery (TDD); virus like nanoparticles (VLPs); water-in-oil (W/O).
